# Nivolumab-induced eosinophilic fasciitis: a case report

**DOI:** 10.1093/rap/rkaa001

**Published:** 2020-01-28

**Authors:** Natasha Ollier, Emilie Tournier, Nicolas Meyer, Vincent Sibaud, Cécile Pages-Laurent, Pierre Cougoul, Odile Beyne-Rauzy, Thibault Comont

**Affiliations:** r1 Service de Médecine Interne, Centre Hospitalier Universitaire de Toulouse, Institut Universitaire du Cancer Toulouse Oncopole; r2 Service d’anatomopathologie, Centre Hospitalier Universitaire de Toulouse, Institut Universitaire du Cancer Toulouse Oncopole; r3 Service d’Oncodermatologie, Institut Universitaire du Cancer Toulouse Oncopole, Toulouse, France

**Key message**

- Eosinophilic fasciitis induced by checkpoint inhibitors needs to be recognized and treated promptly.

Sir, Rheumatic and musculoskeletal immune-related adverse events are relatively rare in cancer patients treated with checkpoint inhibitors [1]. Among them, eosinophilic fasciitis is mentioned in only a few reports. We recently observed an extensive eosinophilic fasciitis after treatment with nivolumab, an antibody targeting programmed death 1 (PD-1).

In 1988, a 35-year-old man was diagnosed with localized superficial spreading melanoma, Breslow 0.8, and was treated by surgical resection with an additional 1-cm-wide margin. In 2014, he underwent a total thyroidectomy and was subsequently treated with radioactive iodine (^131^I) therapy for a papillary carcinoma. In July 2017, he presented with a recurrence of melanoma, with a right pectoral localization. PET–tomodensitometry revealed other metastases: intercostal and pulmonary (stage IV, metastatic stage; M1C). He started treatment with nivolumab in July 2017 at a dosage of 3 mg/kg i.v every 2 weeks. No adverse event was reported initially, and evaluation after 6 and 12 infusions showed a complete metabolic response. The dosage of nivolumab was reduced after 20 infusions; the patient received seven infusions at a fixed dosage (480 mg every month), from April to October 2018.

**Fig. 1:**
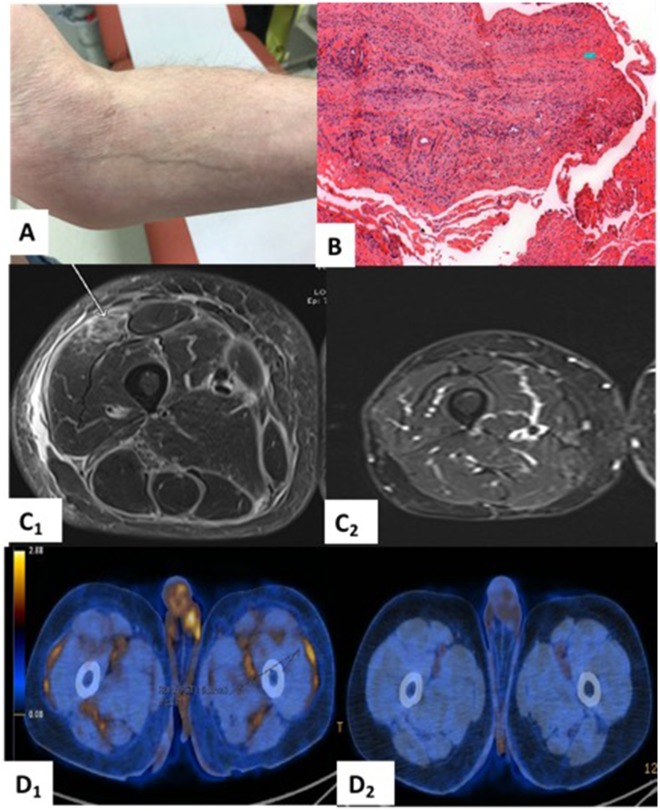
Clinical, morphological and histological features of eosinophilic fasciitis at diagnosis and outcome after treatment (**A**) Groove sign, a linear depression in the skin parallel to the course of the superficial veins. (**B**) Inflammatory infiltrates (lymphocytes, plasmocytes and eosinophils) in the fascia and muscle (leg biopsy, standard coloration, ×10 magnification). (**C**) MRI findings at diagnosis (March 2019; C_1_) and after treatment with MTX and IVIG (December 2019; C_2_) (**D**) PET–tomodensitometry findings at diagnosis (March 2019; D_1_) and after treatment (December 2019; D_2_).

After three cycles of the fixed-dose regimen, he developed diffuse muscular pain, with progressive fatigue and proximal weakness. Clinical examination showed painful, tender and symmetrical oedema of the lower limbs, with subsequent local stiffening of the skin over the back of the thighs and forearms. Symptoms worsened after each infusion of nivolumab, which was discontinued in October 2018 after seven infusions of the fixed-dose regimen. The patient did not report improvement after the discontinuation. In February 2019, physical examination also showed depression along the course of superficial veins (groove sign; Fig. 1A) on the upper limb. His face and fingers were not affected. He also presented with joint pain with limitation of mobility.

Laboratory tests revealed only peripheral eosinophilia of 1800/μl (normal range <500/μl) and inflammatory syndrome (CRP 115 mg/l). Creatine kinase and lactate deshydrogenases rate were normal. Immunological tests were negative. Electroneuromyography was normal. An MRI was performed and showed thickening and enhancement of the fascia in the medial and posterior muscle compartments of the lower limbs (Fig. 1C1). A biopsy of the right thigh was performed and showed fasciitis and myositis, with infiltration of lymphoplasmocytes associated with eosinophils (Fig. 1B). PET–tomodensitometry was also performed (Fig. 1D1), confirming a metabolic response of the melanoma but showing hypermetabolism of the fascias, consistent with eosinophilic fasciitis.

In March 2019, prednisone was started (1 mg/kg p.o. daily), with initial improvement in skin thickening, joint mobility and pain. However, this effect was partial and temporary, and 2 weeks after starting CS (without recurrence of eosinophilia) the symptoms worsened. Weekly MTX was added to prednisone in April 2019 (15 mg/week p.o. with folate on the off days). After 2 months, the patient reported moderate clinical improvement regarding the stiffening of the skin, but in contrast, MRI was significantly improved. The dosage of MTX was increased to 20 mg/week p.o., then 25 mg/week s.c., and IVIG was added (2 g/kg i.v every month). At the last follow-up examination, in December 2019, the patient presented a major clinical improvement, according to morphological findings (Fig. 1C2 and D2).

The most frequent rheumatological immune-related adverse events are arthralgia and myalgia, whereas arthritis, myositis and vasculitis are less reported. Eosinophilic fasciitis is a rare entity and can potentially be induced by checkpoint inhibitors, especially by pembrolizumab [an antibody anti-programmed cell death receptor-1 (PD-L1)] [2, 3] or nivolumab [4]. Treatment often consists of CSs, with a response in the majority of cases [5]. In a recent report published by Toussaint *et al.* [3], the authors described a 77-year-old female patient with acral lentiginous melanoma and with cutaneous and thorax metastases, who developed, after 31 infusions with pembrolizumab, severe myalgia and oedema in the upper arms and thighs, revealing an eosinophilic fasciitis. Her symptoms improved slightly after treatment with prednisolone at a starting dose of 1 mg/kg and slowly tapered. After 2 months, the patient was still on prednisolone at 20 mg/day, and MTX was added at a final dose of 20 mg/week. A complete regression of eosinophilic fasciitis was observed on MRI control after 9 months of this combination therapy. Likewise, our patient did not respond to CSs alone, and we had to add MTX and then IVIG to improve the symptoms.

This case highlights that immune musculoskeletal checkpoint inhibitor toxicities may not respond to CSs alone and that MTX can be used as a second-line treatment. The clinical course may be delayed, and IVIG may be added for transient improvement of efficacy.

*Funding:* No specific funding was received from any funding bodies in the public, commercial or not-for-profit sectors to carry out the work described in this manuscript.

*Disclosure statement:* The authors have declared no conflicts of interest.
